# Variations and Associated Factors in Symptom-to-Balloon (STB) Time and Door-to-Balloon (DTB) Time Before and After the COVID-19 Lockdown: A Hospital-Based Cross-Sectional Study

**DOI:** 10.7759/cureus.47658

**Published:** 2023-10-25

**Authors:** Neeraj V Mohandas, Vijayakumar K, Aswathy Sreedevi, Neethu George, Koshy Eapen, Saji Subramanian, Himal Raj, Jaideep C Menon

**Affiliations:** 1 Community Medicine, Dhanalakshmi Srinivasan Medical College and Hospital, Perambalur, IND; 2 Health Research, Health Action by People, Trivandrum, IND; 3 Community Medicine, Amrita Institute of Medical Sciences, Kochi, IND; 4 Cardiology, Samaritan Heart Institute, Kochi, IND; 5 Cardiology, Amrita Institute of Medical Sciences, Kochi, IND

**Keywords:** covid-19, cad, lockdown, dtb, stb

## Abstract

Introduction: During the COVID-19 lockdown, India saw a major restriction in the movement of people. Patients with acute myocardial infarction (MI) required early interventions and follow-up of independent predictors like symptom-to-balloon (STB) time and door-to-balloon (DTB) time. This study aimed to determine changes in STB and DTB time before and after the COVID-19 lockdown and its associated risk factors.

Methods: A hospital-based cross-sectional study of 105 patients admitted to the cardiac care units (CCU) of two tertiary care centers in a district of Southern India for six months was conducted to compare the changes in STB and DTB time before and after the COVID-19 lockdown (three months before March 2020 and three months after March 2020), and data was collected from medical records. The data collected was then entered into Microsoft Excel (Microsoft Corporation, Washington, USA), numerically coded, and analyzed using SPSS Statistics version 21 (IBM Corp. Released 2012. IBM SPSS Statistics for Windows, Version 21.0. Armonk, NY: IBM Corp.). The Chi-square and Mann-Whitney U tests assessed the association between the dependent and independent variables. The STB/DTB time (before and after the COVID-19 lockdown) was the dependent variable, while the age, gender, co-morbidities, smoking status, and date of admission of patients (before and after the COVID-19 lockdown) were taken as the independent variables. A p-value of <0.05 was considered statistically significant. The predictor variables were identified using the regression method, where all variables with a significance of <0.2 were taken.

Results: The overall mean (±SD) STB time was 408.7 (±307.1) minutes, and the mean (±SD) DTB time was 161.7 (±261.6) minutes. The pre-lockdown mean STB time was 404.6 minutes, and the mean DTB time was 153 minutes, whereas the post-lockdown mean STB and DTB time were higher at 413.3 minutes and 171.6 minutes, respectively. Out of the total 105 patients, 95 (90.5%) had an STB time of ≥120 minutes, and 77 (73.3%) had an ideal DTB time of <90 minutes. There was no statistically significant variation in the STB and DTB time before and after the lockdown. Only the age group >60 years (38 (97.4%)) was found to be statistically significant with an STB time of ≥120 minutes after the lockdown (p-value=0.040), and patients referred from primary and secondary care centers (AOR (95% CI)=4.669 (1.129-19.298)) were found to be an independent factor in reducing DTB time before and after the COVID-19 lockdown.

Conclusion: The efficiency of the health system, irrespective of the COVID-19 lockdown, was observed; nevertheless, a delay in the overall recognition of symptoms of MI was perceived. The importance of time factors in identifying the symptoms of non-communicable diseases (NCDs), especially MI and stroke, has to be ascertained among the general population.

## Introduction

The first national COVID-19 lockdown was declared in India on March 25, 2020, which saw a complete shutdown of all workplaces and transport sectors. Restriction of outside movement, lack of public transport, and social distancing were the major difficulties faced by the public during the COVID-19 lockdown period [1]. Most acute coronary syndrome (ACS) patients in India reach the hospital by public or private transport instead of an ambulance [2]. With the extended phases of the lockdown, most public transport ceased and private vehicles were off the road for varying periods across different states of India. Furthermore, all patients had to undergo the COVID-19 rapid antigen test or RT-PCR before hospital admission as per individual hospital protocols. The COVID-19 testing may have prolonged the definitive management of ST-segment elevation myocardial infarction (STEMI), which the patient should receive at the earliest [3]. Most studies demonstrated that treatment delays to primary percutaneous transluminal coronary angioplasty (PTCA) are associated with increased mortality and larger infarct size in the myocardial tissue [4].

Primary PTCA is the preferred treatment for managing elevated STEMI, which should be done within 12 hours of symptom onset [4-5]. Despite all the advances in the treatment of STEMI, failed myocardial reperfusion occurs in almost 50% of STEMI patients and can be predictive of heart failure in the longer term [4]. Hence, such patients with acute myocardial infarction (MI) require early intervention.

A successful angioplasty combines post-procedural thrombolysis in MI flow grade 3 and residual stenosis of <30% [6]. The treatment delays, which consist of patient delay, the time from symptom onset to first medical contact; prehospital system delay, the time from first medical contact to arrival at a PTCA-capable hospital; and in-hospital delay are important determinants of patient outcome and the most easily audited quality of care index [7].

Studies worldwide showed that the incident rate ratio of all forms of ACS admissions, including STEMI, non-STEMI, and unstable angina, decreased by 30% compared to 2019 [8]. A decrease in the number of primary PTCA and an increase in the time to first medical contact and time to revascularization has been documented [6]. Studies in a large number of countries such as Italy, Austria, Hongkong, England, and the United States showed a significant decrease in acute MI admissions, an increase in the window period, and an increase in out-of-hospital cardiac arrests during the pandemic [9]. On the other hand, the status of acute MI admissions in India during the COVID-19 pandemic period is largely unknown [10].

The concept of symptom-to-balloon (STB) time and time-to-reperfusion, defined as the interval from the onset of symptoms to the first balloon inflation, has been put forth by the American Heart Association (AHA) to emphasize timely management [6]. Hence, STB time is defined as the interval beginning from the episode of chest pain leading the patient to present to the ER to the time of first balloon inflation [11]. Recent studies suggested an ideal STB time of <120 minutes [11]. The door-to-balloon (DTB) time is the time from the arrival at the ED of patients with STEMI until a catheter guidewire crosses the culprit lesion in the cardiac catheterization lab [11]. A DTB time of ≤90 minutes for at least 85% of patients is a well-established cardiovascular goal for patients with STEMI undergoing PTCA [11].

The study aimed to determine the changes in the STB and DTB time before and after the COVID-19 lockdown and assess the determinants of variation in the STB time during the same time period.

## Materials and methods

A hospital-based cross-sectional study was conducted among patients in two tertiary care hospitals with cardiac care units (CCU) in Ernakulam District of Kerala State in South India for six months (three months before March 2020 and three months after March 2020). Institutional Ethics Committee clearance from Amrita Institute of Medical Sciences was obtained prior to the start of the study (approval no. IEC-AIMS-2021-COMM-029). A total of 105 patients were taken for the study.

Sample size

All patients diagnosed with acute MI on whom primary PTCA was done during the study period were included in the study from the selected two hospitals, numbering 110 (60 before lockdown and 50 after lockdown). Five patients with incomplete medical records were excluded from both hospitals. Finally, 56 and 49 patients from the two hospitals were taken before and after the lockdown, respectively, for the analysis.

Diagnostic criteria

Diagnosis of ACS was defined as a combination of ECG criteria and elevation of cardiac biomarkers according to the fourth universal definition of MI [12]. The patients fitting the above criteria were included in the study. In MI, three main coronary arteries are involved: the right coronary artery, the left anterior descending artery, and the left circumflex artery [13]. The symptoms and the prognosis of MI are proportional to the number of vessels affected. The ischemic burden is maximum for multi-vessel disease (including two and three vessel disease) and least for single vessel disease [13-14].

Sampling technique

Data was collected from the medical records of the patients accessed through the medical records departments of both hospitals after obtaining permission from the hospital administrative authorities. A pretested, semi-structured questionnaire was used to collect data from the medical records. This included details of sociodemographic variables, history of co-morbidities, personal habits such as tobacco use, referral, and PTCA details performed such as the date and time of onset of chest pain, date and time the patient reached the hospital, and when the patient was shifted to the cardiac catheterization laboratory for primary PTCA.

Statistical analysis

The data collected was then entered into Microsoft Excel (Microsoft Corporation, Washington, USA), numerically coded, and analyzed using SPSS Statistics version 21 (IBM Corp. Released 2012. IBM SPSS Statistics for Windows, Version 21.0. Armonk, NY: IBM Corp.). The Chi-square and Mann-Whitney U tests assessed the association between the dependent and independent variables. A p-value of <0.05 was considered statistically significant. The predictor variables were identified using a regression model where all variables with a significance of <0.2 were taken.

## Results

The mean age of the 105 study participants (56 before and 49 after the lockdown) was 56±10.3 years. The study participants in the age group under 60 were 72 (68.5%). Majority of the study participants were males, 88 (83.8%). The overall median (IQR) STB time was 300 minutes (190, 557.5). The STB time increased from a median of 280 minutes (177.25, 562.5) before the lockdown to 360 minutes (190, 572.5) after the lockdown. Table 1 shows additional details.

**Table 1:** Distribution of the profile of the study participants in STB *Multiple responses

Variables		Frequency (n=105)	Percentage (%)
Age (years)	≤60	72	68.5
	>60	33	31.5
Gender	Male	88	83.8
	Female	17	16.2
Co-morbidities*	Diabetes mellitus	50	47.6
	Hypertension	43	41
	Dyslipidemia	42	40
Smoking	Yes	37	35.2
	No	68	64.8

The median STB time and DTB time were higher after the lockdown. However, compared to the median, the difference was not statistically significant (Table 2).

**Table 2:** Variation in STB and DTB time before and after the COVID-19 lockdown Mann-Whitney U test represented as median (IQR). STB: symptom-to-balloon, DTB: door-to-balloon time ^*^Total number of patients taken before the COVID-19 lockdown ^#^Total number of patients taken after the COVID-19 lockdown

Variables		Median (IQR) (minutes)	p-value
STB time (minutes)	Before COVID-19 lockdown (56)*	280 (177.25, 562.5)	0.587
	After COVID-19 lockdown (49)^#^	360 (190, 572.5)	
DTB time (minutes)	Before COVID-19 lockdown (56)*	50 (30, 83.75)	0.266
	After COVID-19 lockdown (49)^#^	60 (33.5, 192.5)	

Before and after the lockdown period, the STB time changes were assessed as not statistically significant (p-value=0.259). The changes in DTB time before and after the lockdown period also showed no statistical significance (p-value=0.392) (Table 3).

**Table 3:** Changes in STB and DTB time before and after the COVID-19 lockdown STB: symptom-to-balloon, DTB: door-to-balloon time, MI: myocardial infarction

Variable		Admission due to acute MI		ꭓ2-value	p-value
		Before lockdown n (%)	After lockdown n (%)		
STB	<120 min	7 (12.5)	3 (6.1)	1.27	0.259
	≥120 min	49 (87.5)	46 (93.9)		
DTB	≤90 min	43 (76.8)	34 (69.4)	0.731	0.392
	>90 min	13 (23.2)	15 (30.6)		

The factors associated with the STB time before and after the lockdown were studied, and only age >60 years was found to be significantly associated with an STB time of ≥120 minutes after the lockdown (p-value=0.040) (Table 4).

**Table 4:** Factors associated with STB time before and after the COVID-19 lockdown *p-value of <0.05 is significant ^#^VD: vessel disease (explained in methods)

Variable		STB time before COVID-19 lockdown		p-value	STB time after COVID-19 lockdown		p-value
		<120 min n (%)	≥120 min n (%)		<120 min n (%)	≥120 min n (%)	
Age (years)	≤60	2 (10)	18 (90)	0.673	2 (20)	8 (80)	0.040*
	>60	5 (13.9)	31 (86.1)		1 (2.6)	38 (97.4)	
Gender	Male	6 (13.3)	39 (86.7)	0.703	3 (7)	40 (93)	0.504
	Female	1 (9.1)	10 (90.9)		0 (0)	6 (100)	
Smoking	Yes	0 (0)	15 (100)	0.087	2 (9.1)	20 (90.9)	0.434
	No	7 (17.1)	34 (82.9)		1 (3.7)	26 (96.3)	
Patient referred from primary and secondary care centers	Yes	1 (5)	19 (95)	0.206	2 (7.7)	24 (92.3)	0.626
	No	6 (16.7)	30 (83.3)		1 (4.3)	22 (95.7)	
Number of coronary arteries involved	SVD^#^	1 (5.3)	18 (94.7)	0.175	0 (0)	9 (100)	0.482
	2VD^#^	1 (6.7)	14 (93.3)		2 (11.1)	16 (88.9)	
	3VD^#^	5 (22.7)	17 (77.3)		1 (4.5)	21 (95.5)	

The factors associated with the DTB time before and after the lockdown were studied, and referred cases from the secondary care level were found to be significantly associated with the DTB time before and after the lockdown (p-value=0.016, p-value=0.014, respectively) (Table 5).

**Table 5:** Factors associated with the DTB time before and after the COVID-19 lockdown *p-value of <0.05 is significant ^#^VD: vessel disease (explained in methods)

Variable		DTB time before COVID-19 lockdown		p-value	DTB time after COVID-19 lockdown		p-value
		≤90 min n (%)	>90 min n (%)		≤90 min n (%)	>90 min n (%)	
Age (years)	≤60	16 (80)	4 (20)	0.671	6 (60)	4 (40)	0.470
	>60	27 (75)	9 (25)		28 (71.8)	11 (28.2)	
Gender	Male	34 (75.6)	11 (24.4)	0.659	29 (67.4)	14 (32.6)	0.429
	Female	9 (81.8)	2 (18.2)		5 (83.3)	1 (16.7)	
Smoking	Yes	12 (80)	3 (20)	0.730	15 (68.2)	7 (31.8)	0.869
	No	31 (75.6)	10 (24.4)		19 (70.4)	8 (29.6)	
Patient referred from primary and secondary care centers	Yes	19 (95)	1 (5)	0.016*	22 (84.6)	4 (15.4)	0.014*
	No	24 (66.7)	12 (33.3)		12 (52.2)	11 (47.8)	
Number of coronary arteries involved	SVD^#^	16 (84.2)	3 (15.8)	0.452	4 (44.4)	5 (55.6)	0.187
	2VD^#^	12 (80)	3 (20)		14 (77.8)	4 (22.2)	
	3VD^#^	15 (68.2)	7 (31.8)		16 (72.7)	6 (27.3)	

Binomial logistic regression was done for the DTB time after the COVID-19 lockdown with significant predictor variables from univariate analysis. Analyses revealed a good model fit (discrimination among groups) based on the presence of patient referrals from primary and secondary care centers and the number of coronary arteries involved (χ2-value=1.756, p-value=0.781). The subjects not referred have 4.6 times higher odds of having a higher DTB time after the COVID-19 lockdown (Table 6).

**Table 6:** Independent factors for the delay in the DTB time after the COVID-19 lockdown *p-value of <0.05 is significant ^#^VD: vessel disease (explained in methods)

Sl. No.	Variables		DTB time after COVID-19 lockdown		Adjusted OR (95% CI)	p-value
			≤90 min n (%)	>90 min n (%)		
1.	Patient referred from primary and secondary care centers	Yes	22 (84.6)	4 (15.4)	1	0.033*
		No	12 (52.2)	11 (47.8)	4.669 (1.129-19.298)	
2.	Number of coronary arteries involved	3VD^#^	4 (44.4)	5 (55.6)	1	0.393
		2VD^#^	14 (77.8)	4 (22.2)	1.163 (0.239-5.669)	
		SVD^#^	16 (72.7)	6 (27.3)	3.197 (0.575-17.684)	

## Discussion

This study focuses on the STB time, the DTB time, and changes during the COVID-19 lockdown period. No significant changes were seen in the STB and DTB time before and after the COVID-19 lockdown period (p=0.259 and p=0.392, respectively).

The STB and DTB time estimate total ischemic time, which is critical for myocardial salvage and survival. All the patients presenting to the ED (Emergency Department) with symptoms of ACS needed time-specific care [15]. In this study, the majority of the patients (73.3%) got a DTB time of ≤90 minutes and the majority of the patients (90.5%) got an STB time of ≥120 minutes despite the COVID-19 lockdown. However, according to the AHA and the American College of Cardiology guidelines, the DTB time should be within 90 minutes for 85% of STEMI patients [16]. A recent study conducted in Japan showed that the DTB time was longer in patients after the COVID-19 lockdown [17]. The reason can mostly be attributed to the extra time taken for the COVID-19 testing to be done.

Studies showed an STB time of ≥120 minutes is associated with increased infarct size, increased transmurality index, and decreased myocardial salvage index [4]. Recent studies by Greulich et al. using late gadolinium enhancement cardiac MRI after PTCA demonstrated that the extent of salvaged myocardium is maximum when reperfusion is obtained within 90-120 minutes of symptom onset [5].

In this study, there were no significant changes in the STB and DTB time before and after the lockdown (p=0.36), which denotes the efficiency of the health system despite the COVID-19 lockdown. However, there was an overall delay in the STB time, irrespective of the lockdown, which needed to be addressed.

This study had a statistically significant association between DTB time and patients referred from primary and secondary healthcare centers before and after the COVID-19 lockdown (p-value=0.016 and p-value=0.014, respectively). The patients not referred had 4.6 times the odds of delay in receiving an ideal DTB time, even though it was not statistically significant. A study done in Chicago demonstrated that establishing a STEMI referral program between a secondary and tertiary care hospital with a cardiac catheterization lab facility would markedly improve the time to reperfusion [18].

This study observed a statistically significant association between age and STB time after the COVID-19 lockdown (p-value=0.040), which could be because most participants, especially the elderly population, had difficulties reaching hospitals during the lockdown period. A study by Mahapatra et al. focused on the difficulties in transport and anxiety the elderly population had during the COVID-19 pandemic [19].

In an ideal system of cardiac care, patients should be able to recognize symptoms and contact emergency services promptly to benefit most from reperfusion therapy. However, recent studies suggested that the inability to recognize and describe somatic feelings appropriately influences STEMI patients, leading to a longer delay in calling for medical help, which can be seen in the overall increase in the STB time despite the lockdown [20]. Innovative and multidisciplinary research in this field should be taken up to pave the way to novel strategies for motivating and educating patients to contact emergency services promptly so that the ischemic time can be further shortened [21]. The outcomes of this could be the basis for further research to determine other variants and risk factors leading to the delay in time to reperfusion in a typical clinical scenario.

There are a few limitations in this study. Patients from only two tertiary care hospitals were taken from the district. The distance from the place of residence to the hospital, patients' health literacy and literacy status, and their socioeconomic status could not be assessed as that data was not available in the medical records. There is a possibility of misclassification bias where the time (STB and DTB) may not be accurate as it was taken from the medical records.

## Conclusions

This study concludes that there are no significant changes in the STB and DTB time during the COVID-19 lockdown. However, it also concludes that very few patients get an ideal STB, which means there is a need to improve the early detection of MI. The perception of people regarding the importance of response time in infarction should be improved via health education. Most patients get an ideal DTB time, and the patients referred from primary and secondary care centers after preliminary diagnosis were found to be an independent predictor for reducing the DTB time. Therefore, strengthening the first contact in the health care system is crucial as it improves the response time and the efficiency of the tertiary health system as a whole.
